# Exploring the boundaries of Niemann-Pick disease type A/B: a report of a case and review of literature

**DOI:** 10.1186/s40348-025-00206-z

**Published:** 2025-11-10

**Authors:** Mohamed El-mezayen, Abdelrahman M. Tawfik, Abdalla M. Hadhoud, Virginia M. Gerges, Mohamed H. Afify

**Affiliations:** Faculty of Medicine, Alexandria University, Alexandria, Egypt

**Keywords:** Acid sphingomyelinase deficiency, Niemann-Pick disease, Meningoencephalitis, Seizures, Lysosomal storage disorder, Inborn error of metabolism, SMPD1, Cherry-red spot, Case report

## Abstract

**Background:**

Acid sphingomyelinase deficiency (ASMD), also known as Niemann-Pick disease types A and B, is a rare autosomal recessive lysosomal storage disorder caused by *SMPD1* mutations. It is characterized by sphingomyelin accumulation and a broad clinical spectrum ranging from severe neurodegeneration in type A to a milder visceral phenotype in type B. Intermediate forms (type A/B) show overlapping features of both subtypes.

**Case presentation:**

We report a 6-month-old boy with ASMD type A/B who first presented with meningoencephalitis and a single seizure most likely secondary to an intercurrent viral infection rather than a primary disease manifestation. Subsequent evaluation revealed multiple systemic red-flag features including marked hepatosplenomegaly, severe growth failure, a cherry-red spot, macroglossia, dysmorphic facial features, recurrent pneumonia, and bilateral sensorineural hearing loss. Laboratory investigations demonstrated elevated liver enzymes and cerebrospinal fluid abnormalities, while auditory brainstem response confirmed the hearing impairment. Enzyme assay confirmed reduced ASM activity, and targeted *SMPD1* genetic sequencing identified a homozygous frameshift mutation classified as pathogenic according to ACMG criteria, establishing the diagnosis of an intermediate ASMD phenotype.

**Conclusion:**

This case highlights the diagnostic challenges posed by ASMD type A/B, particularly when the initial presentation mimics an acute infection. The overlap of coincidental infectious illness with systemic red-flag features, the clinical variability of intermediate phenotypes, and the rarity of the disorder all contribute to delayed recognition. These factors underscore the importance of maintaining a high index of suspicion and pursuing early metabolic and genetic testing in atypical pediatric presentations.

**Trial registration:**

Not applicable.

**Supplementary Information:**

The online version contains supplementary material available at 10.1186/s40348-025-00206-z.

## Background

Niemann-Pick disease type A/B (NPD A/B), also known as acid sphingomyelinase deficiency (ASMD; OMIM #257200, #607616), is a rare inborn error of metabolism and lysosomal storage disorder characterized by deficient activity of the acid sphingomyelinase (ASM) enzyme, resulting in the accumulation of sphingomyelin and other lipids in various tissues [1]. The disease has an estimated incidence of approximately 1 in 250,000 individuals, with a higher prevalence among Ashkenazi Jewish populations [1, 2]. Clinically, NPD A/B presents in two primary subtypes: type A, a severe infantile neurovisceral form with rapid neurological deterioration and death typically by age 2–3 years; and type B, a milder form with predominantly visceral involvement, minimal neurological symptoms, and survival often into adulthood. Intermediate phenotypes, exhibiting features of both subtypes, have also been reported, highlighting the disease’s clinical heterogeneity [1, 3, 4].

NPD A/B is inherited as an autosomal recessive disorder caused by mutations in the *SMPD1* gene, located on chromosome 11p15.4, which encodes ASM. Over 180 mutations have been identified, contributing to the variable clinical spectrum [1, 4]. Pathophysiologically, ASM deficiency leads to the accumulation of sphingomyelin and its precursors within lysosomes, particularly in macrophages, forming lipid-laden foam cells that deposit in organs such as the liver, spleen, lungs, and, in type A, the central nervous system. This accumulation results in hepatosplenomegaly, cytopenias, interstitial lung disease, and, in severe cases, progressive neurodegeneration [1, 5].

Diagnosis is typically achieved by measuring ASM activity in leukocytes, with genetic sequencing of the *SMPD1* gene for confirmation. Recent advances include dried blood spot assays for enzymatic activity, enhancing diagnostic accessibility [1]. Early recognition of NPD A/B is critical. The timely diagnosis facilitates supportive care and access to emerging therapies such as enzyme replacement therapy with olipudase alfa, which has shown promise in reducing organ volumes and improving lipid profiles [3, 6]. The rarity and clinical variability of NPD A/B often lead to diagnostic delays, as illustrated by cases initially misdiagnosed as Gaucher disease [3].

Reporting cases with intermediate phenotypes is particularly valuable, as these expand the understanding of the disease spectrum, aid in accurate diagnosis, and inform the development of tailored therapeutic strategies [4]. This case report of a 6-month-old boy with NPD A/B underscores the importance of recognizing atypical presentations specifically, an infection-like onset with meningoencephalitis and seizure when accompanied by systemic features such as hepatosplenomegaly, growth failure, and a cherry-red spot, which ultimately pointed toward the correct metabolic diagnosis.

## Case presentation

A 6-month-old boy was admitted to Alexandria Main University Hospital, Alexandria, Egypt in May 2024 with symptoms of fever, vomiting, and hypoactivity. He was born at term via spontaneous vaginal delivery after an uneventful pregnancy, with a birth weight of 3.2 kg (25th–50th percentile) and no perinatal complications. His developmental milestones were slightly delayed: he achieved partial head control by 4 months but had not yet rolled over or begun social smiling at 6 months.

The family history was notable for hereditary and hematological conditions: the patient’s uncle and grandfather were suspected of hereditary spastic myelopathy, a cousin was diagnosed with thalassemia, and his mother suffered from rheumatoid arthritis. She also experienced two miscarriages before this child. No consanguinity was reported.

On admission, cerebrospinal fluid (CSF) analysis revealed elevated white cells, with a polymorphonuclear count of 48 cells/µL and lymphocytes at 84 cells/µL, suggestive of central nervous system infection. Blood cultures were negative without evidence of organism growth. Initial blood work showed a hemoglobin level of 9.6 g/dL, white blood cell count of 10,000/µL, and platelets at 300,000/µL. Liver enzymes were elevated (AST: 220 U/L and ALT: 112 U/L). A chronological description of the laboratory findings is mentioned in (Table 1). Physical examination revealed hepatosplenomegaly and minimal abdominopelvic ascites.

**Table 1:** Chronological description of the laboratory findings at multiple admissions

		May 2024	June 2024	December 2024	February 2025	March 2025	Reference Range
**Haematology**							
*WBCs*	*B%*		1.5	0.3		0	*0–1%*
	*E%*		1.9	0.4		3	*1–5%*
	*N%*		26.9	55.4		38	*20–50%*
	*L%*		53.2	38.1		52	*40–70%*
	*M%*		4.5	5.2		7	*2–10%*
	*Total*	10	15.6	15.49	18	7.8	*6.0–17.5 × 10³/μL*
*Hb*		9.6	12	10.4		9.8	*9.5–14.0 g/dL*
*Ht*			37.7	33		29.4	*28–40%*
*MCV*			76.4	74.5		70	*74–86 fL*
*MCH*			24.3	23.5		23.4	*25–31 pg*
*MCHC*			31.8	31.5		33.3	*30–35 g/dL*
*Plt*		300	484	267		188	*150–450 × 10³/μL*
**Renal Function**							
*BUN*		9	5	8			*5–18 mg/dL*
*Creatinine*		0.4	0.3	0.18	0.6		*0.2–0.6 mg/dL*
*Urea*			11	17			*5–18 mg/dL*
**Liver Function**							
*ALT*		52	161		77	67	*5–50 U/L*
*AST*		119	517		176	221	*15–65 U/L*
*ALP*			273				*150–500 U/L*
*TSB*			0.5				*0.1–1.2 mg/dL*
*DSB*			0.2				*0–0.4 mg/dL*
*Albumin*			3.3	3.9			*3.5–5.0 g/dL*
**Electrolytes**							
*Na*			139	136	130	141	*135–145 mEq/L*
*K*			4.3	4.6	5.4	4	*3.5–5.5 mEq/L*
*Cl*			103	102	109		*98–107 mEq/L*
*Ca*			8.6	9.1		11.89	*8.8–11.3 mg/dL*
*P*			3.5	3.6			*4.5–7.5 mg/dL*
**Lipid Profile**							
*TC*			385			246	*100–190 mg/dL*
*TG*			590			373	*30–90 mg/dL*
**Coagulation profile**							
*PT*		12.3	11.9				*11–14 sec*
*INR*		1	1.08				*0.9–1.2*
**Inflammatory marker**							
* CRP*		8.7	3.8				*<1.0 mg/dL *

*Albumin* Serum Albumin, *ALP* Alkaline Phosphatase, *ALT* Alanine Aminotransferase, *AST* Aspartate Aminotransferase, *B%* Basophils Percentage, *BUN* Blood Urea Nitrogen, *Ca* Calcium, *Cl* Chloride, *CRP* C-Reactive Protein, *Creatinine* Serum Creatinine, *DSB* Direct Serum Bilirubin, *E%* Eosinophils Percentage, *Hb* Hemoglobin, *Ht* Hematocrit, *INR* International Normalized Ratio, *K* Potassium, *L%* Lymphocytes Percentage, *M%* Monocytes Percentage, *MCH* Mean Corpuscular Hemoglobin, *MCHC* Mean Corpuscular Hemoglobin Concentration, *MCV* Mean Corpuscular Volume, *Na* Sodium, *N%* Neutrophils Percentage, *P* Phosphorus, *Plt* Platelets, *PT* Prothrombin Time, *RBCs* Red Blood Cells, *TSB* Total Serum Bilirubin, *TC* Total Cholesterol, *TG* Triglycerides, *WBC Total* Total White Blood Cells

During the course of this illness, the patient experienced a single generalized seizure lasting approximately two minutes, followed by a postictal period of drowsiness. There was no prior history of seizures, and no chronic antiepileptic medication was initiated, as the seizure was considered to be secondary to acute CNS infection. A diagnosis of meningoencephalitis was made, and the patient started on empirical treatment, including antibiotics, acyclovir, dexamethasone, and supportive care. The child showed gradual clinical improvement, regaining full orientation and activity, with normalized feeding and stable hemodynamics. Liver enzymes began to decline with clinical recovery (AST: 11 U/L and ALT: 52 U/L).

A month later, the patient was readmitted with fever and illness. Abdominal ultrasound confirmed hepatosplenomegaly, with the liver measuring 9.5 cm and spleen 7.5 cm, both with preserved parenchymal patterns (Supplementary Figure.1). Sickle cell disease was suspected but then excluded after Hemoglobin electrophoresis (HbA1: 96.5%, HbA2: 2.7%, and HbF: 0.8%). MRI of the brain with contrast was performed during this admission and showed no abnormalities as demonstrated in (Supplementary Figures.2).

Given the combination of organomegaly and a prior central nervous system event, a lysosomal storage disorder was strongly suspected, with Gaucher disease and Niemann-Pick disease type A/B considered as differential diagnoses. Screening for lysosphingomyelin (Lyso-SPM) revealed a markedly elevated level of 601.2 ng/mL. Targeted next-generation sequencing using a lysosomal storage disorder gene panel identified homozygous frameshift mutations in *SMPD1* (NM_000543.5: c.685G > T; p.Gly229Cysfs*?), classified as pathogenic according to ACMG criteria. Parental genetic testing was not performed due to logistical constraints. The identified variant has been rarely reported in the Egyptian population and is not considered a founder mutation. Enzyme assay by tandem mass spectrometry confirmed markedly reduced acid sphingomyelinase activity, thereby establishing the diagnosis of acid sphingomyelinase deficiency (ASMD). In contrast, β-glucocerebrosidase activity was within the normal range, effectively excluding Gaucher disease.

Due to the previous episode of meningoencephalitis, an Auditory Brainstem Response (ABR) test was conducted. It revealed a right-sided severe and left-sided mild sensorineural hearing loss, affecting the mid to high-frequency range (2000–4000 Hz) (Supplementary Figure.3).

Two months later, the patient was readmitted for neurological concerns including a bulging anterior fontanelle. On examination, characteristic features such as a cherry-red spot on fundus exam, macroglossia, hepatosplenomegaly, low-set ears, and a depressed nasal bridge were observed—further supporting a lysosomal storage disorder phenotype. A repeat CSF analysis showed elevated protein (148 mg/dL) with normal glucose and low cellularity; cultures remained negative.

**Fig. 1:**
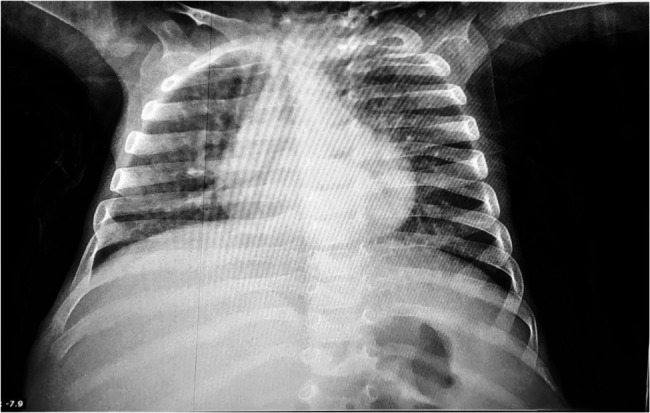
Showing the patient’s X-ray during his second pneumonia episode

Over the following 6 months, the patient experienced recurrent respiratory infections, including two episodes of pneumonia. The first was characterized by fever, respiratory distress, bilateral crepitations, and abnormal findings on chest X-ray. The second pneumonia episode was more severe, presenting with cyanosis and hypoxia (SpO₂ 89%) and right patchy infiltrates on imaging (Fig. 1). Laboratory tests showed hyponatremia (130 mmol/L), hyperkalemia (5.4 mmol/L), hypercalcemia (10.9 mmol/L), and liver enzyme elevation (AST: 176 U/L, ALT: 77 U/L), with normal kidney function.

**Fig. 2:**
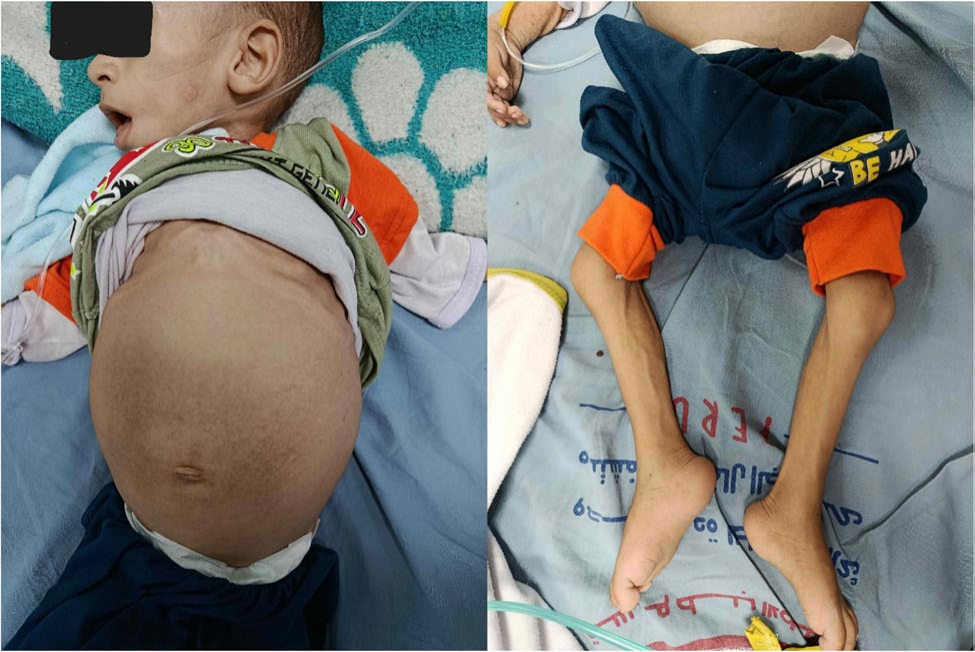
Showing the patient’s clinical appearance manifesting a massive abdominal distention and muscle wasting of the limbs

**Fig. 3:**
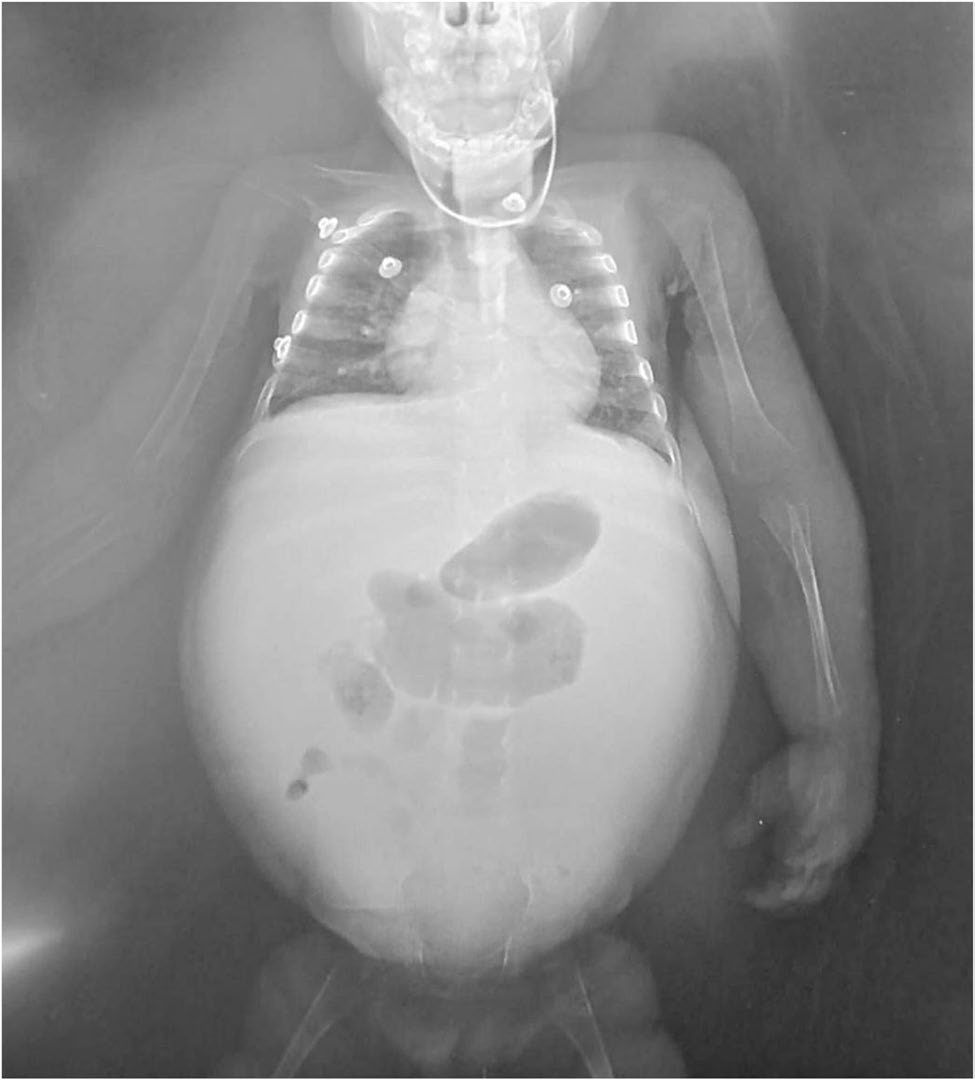
Abdominal X-ray picture showing abdominal distension with dilated bowel loops

His anthropometric measurements reveal significant growth failure, with a weight of 6 kg and length of 75 cm at 17 months of age, both falling below the 5th percentile. The clinical appearance of the patient reflects poor weight gain including notably thin limbs indicative of muscle wasting and possible inadequate protein and calorie intake, pale and potentially dry skin and prominent veins on the extremities suggesting low body fat and overall undernourishment. Moreover, the patient clinically suffers from severe abdominal distention, likely due to severe hepatosplenomegaly (Fig. 2). This finding is further elucidated by an abdominal X-ray of a showing massive abdominal distension with dilated bowel loops and displacement of the diaphragm, and the lungs are compressed, indicating increased intra-abdominal pressure. (Fig. 3).

## Discussion

ASMD is a rare lysosomal storage disorder resulting from mutations in the *SMPD1* gene, leading to deficient ASM activity and subsequent sphingomyelin accumulation in multiple tissues [7]. The present case of a 6-month-old boy with ASMD type A/B, presenting with meningoencephalitis, seizures, hepatosplenomegaly (liver 9.5 cm, spleen 7.5 cm), growth failure (weight 6 kg, length 75 cm), cherry-red spot, macroglossia, dysmorphic features (low-set ears, depressed nasal bridge), recurrent pneumonia, and sensorineural hearing loss, offers a unique opportunity to compare this case with other cases reported in the literature. (Table 2) mentions the clinical findings and features of this case and compares it with levels of prevalence reported in the current body of literature. Although neurological involvement is typical in ASMD type A, the acute presentation with meningoencephalitis in this patient was most likely coincidental and due to a viral etiology. The subsequent seizure was considered a secondary event. This distinction is important because attributing intercurrent infections to the primary disease may mislead clinicians.

**Table 2:** Clinical features comparison in ASMD type A/B

Feature	Present Case	Literature Prevalence
Meningoencephalitis	Yes	Rare [8]
Seizures	Yes	Uncommon initially [9]
Hepatosplenomegaly	Yes	73–78% [10]
Growth Failure	Yes	Common in type A [8]
Cherry-Red Spot	Yes	Nearly 100% in type A, 33% in type B [9]
Macroglossia	Yes	Rare [10]
Dysmorphic Features	Yes	Less common [10]
Recurrent Pneumonia	Yes	Common [10]
Sensorineural Hearing Loss	Yes	Noted in some cases [10]

The initial presentation with meningoencephalitis, evidenced by CSF analysis showing elevated white cells (polymorphonuclear count 48 cells/*µ*L, lymphocytes 84 cells/*µ*L) and later elevated protein (148 mg/dL), is highly unusual for ASMD. While neurological involvement is a hallmark of type A, characterized by developmental delay, ataxia, and cognitive decline, meningoencephalitis is rarely reported as an initial feature [7, 8]. Seizures are noted as a complication in type A but typically manifest later in the disease course [9]. A prospective study of 64 ASMD type B patients found that 30% exhibited neurological abnormalities, but seizures were not highlighted as an initial presentation [10]. The single generalized seizure in this case, lasting approximately two minutes, was considered secondary to the CNS infection, and the absence of chronic antiepileptic medication aligns with this acute context.

What made this case diagnostically significant was not the episode of meningoencephalitis itself—likely an intercurrent viral illness—but the co-presence of additional systemic signs. Severe hepatosplenomegaly, growth failure evident before the second year of life, cherry-red spot, dysmorphic features, macroglossia, and sensorineural hearing loss all pointed toward an underlying lysosomal storage disorder. The convergence of these features, in the context of an infection-like presentation, highlights the importance of maintaining a high index of suspicion for ASMD and related conditions.

Hepatosplenomegaly is a common early finding in both type A and type B, with 78% and 73% of patients presenting with splenomegaly and hepatomegaly, respectively [10]. Growth failure is a hallmark of type A, often evident by the second year of life [11]. The cherry-red spot is nearly universal in type A by 12 months but less common in type B [9]. Recurrent pneumonia is frequent in type A due to aspiration secondary to neurological impairment and in type B due to pulmonary involvement [10]. Additionally, limited chest expansion die to hepatosplenomegaly is a contributing factor to pulmonary affection. Sensorineural hearing loss, while less frequently reported, has been noted in cases with neurological involvement. Macroglossia and dysmorphic features are less commonly documented but have been reported in severe type A cases [10].

Elevated liver enzymes, particularly alanine aminotransferase and aspartate aminotransferase, are common in ASMD, with up to 75% of patients showing raised transaminases [9, 10]. The ABR revealed right-sided severe and left-sided mild sensorineural hearing loss, affecting the mid to high-frequency range (2000–4000 Hz). Specific ABR findings are not extensively documented in ASMD type A/B literature. The patient’s weight (6 kg) and length (75 cm), both below the 5th percentile, are consistent with the severe growth failure seen in type A/B [8, 10].

ASMD results from mutations in the *SMPD1* gene. The patient had a homozygous frameshift mutation, which typically leads to a severe phenotype. The classification as type A/B suggests some residual ASM activity. The ∆R608 mutation is exclusive to type B, while Q292K is associated with intermediate phenotype [7, 10]. A359D is common in Chilean type B patients [7]. The frameshift mutation in this case likely contributes to the severe phenotype, though residual enzyme function may explain the intermediate type A/B features [12].

Neiman-Pick disease could be misdiagnosed as Gaucher disease due to some similar features such as hepatosplenomegaly, but the latter can be distinguished by bone involvement and β-Glucocerebrosidase accumulation [13]. On the other hand, Tay-Sachs disease shares similar neurological symptoms and the cherry-red spot with ASMD but lacks hepatosplenomegaly [14]. (Supplementary Table 1) shows the main differences between the three differential diagnoses.

Enzyme replacement therapy (ERT) with olipudase alfa improves visceral symptoms but does not cross the blood-brain barrier [10, 15]. Supportive care includes physiotherapy, nutritional support, seizure control, and respiratory care [10]. Emerging therapies such as gene therapy and substrate reduction therapies are currently under investigation [7].

Earlier diagnosis through newborn screening and early ASM activity measurement can guide early ERT initiation [16]. A multidisciplinary care approach with regular neurological, hepatic, and respiratory assessments is essential [10]. Future research should prioritize CNS-penetrant therapies and long-term outcome data [12]. 

## Conclusion

This case of ASMD type A/B with an initial presentation of meningoencephalitis and seizures highlights phenotypic variability. Classical features such as hepatosplenomegaly and cherry-red spots were present alongside less typical findings like macroglossia and dysmorphism. A homozygous frameshift mutation likely explains the intermediate phenotype. Importantly, even in the setting of an intercurrent infection, additional clinical findings should raise a high index of suspicion for this rare disease, emphasizing the need for early recognition and progress toward CNS-directed therapies.

## Supplementary Information

Supplementary Material 1.

## Data Availability

The data used to write this report is available from the corresponding author on reasonable request.

## Abbreviations

ASMD: Acid sphingomyelinase deficiency
ASM: Acid sphingomyelinase
NPD A/B: Niemann-Pick disease type A/B
CSF: Cerebrospinal fluid
Lyso SPM: Lysosomal sphingomyelin storage material
ERT: Enzyme replacement therapy
