# Supplementary material for: Exploring the boundaries of Niemann-Pick disease type A/B: a report of a case and review of literature
Source: Mol Cell Pediatr. 2025 Nov 10;12:18. doi: 10.1186/s40348-025-00206-z (PMC12597853; doi:10.1186/s40348-025-00206-z)
Supplement: Supplementary file 1 — Supplementary Material 1. [file 40348_2025_206_MOESM1_ESM.docx]

**Supplementary Material Legend**

- **Supplementary Figure.1 –** (Abdominal ultrasound showing hepatosplenomegaly)
- **Supplementary Figure.2 – (**Sagittal views of patient’s brain MRI with contrast)
- **Supplementary Figure.3 –** (Evoked potential report of the auditory brainstem response test)
- **Supplementary Table.1 -** (Differential Diagnosis of ASMD Type A/B)


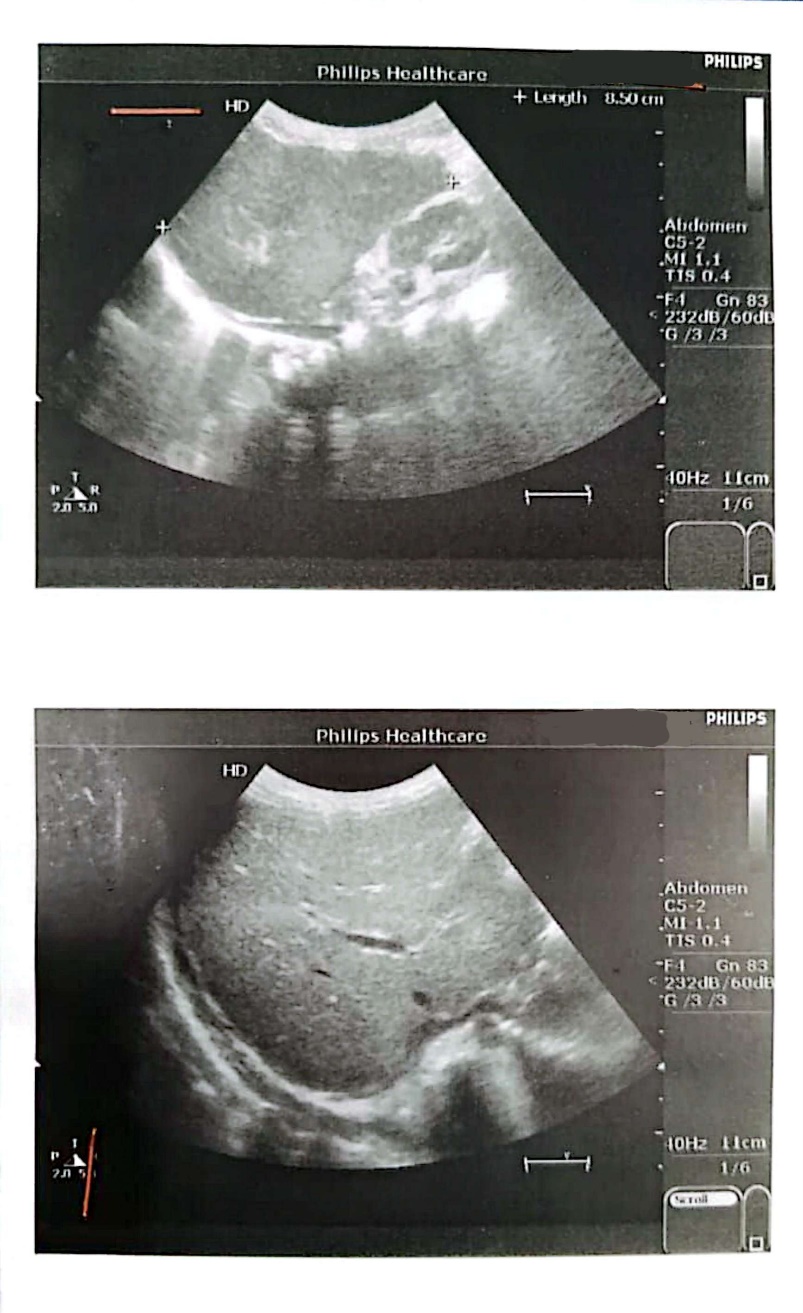


**Supplementary Figure.1 –** (Abdominal ultrasound showing hepatosplenomegaly)


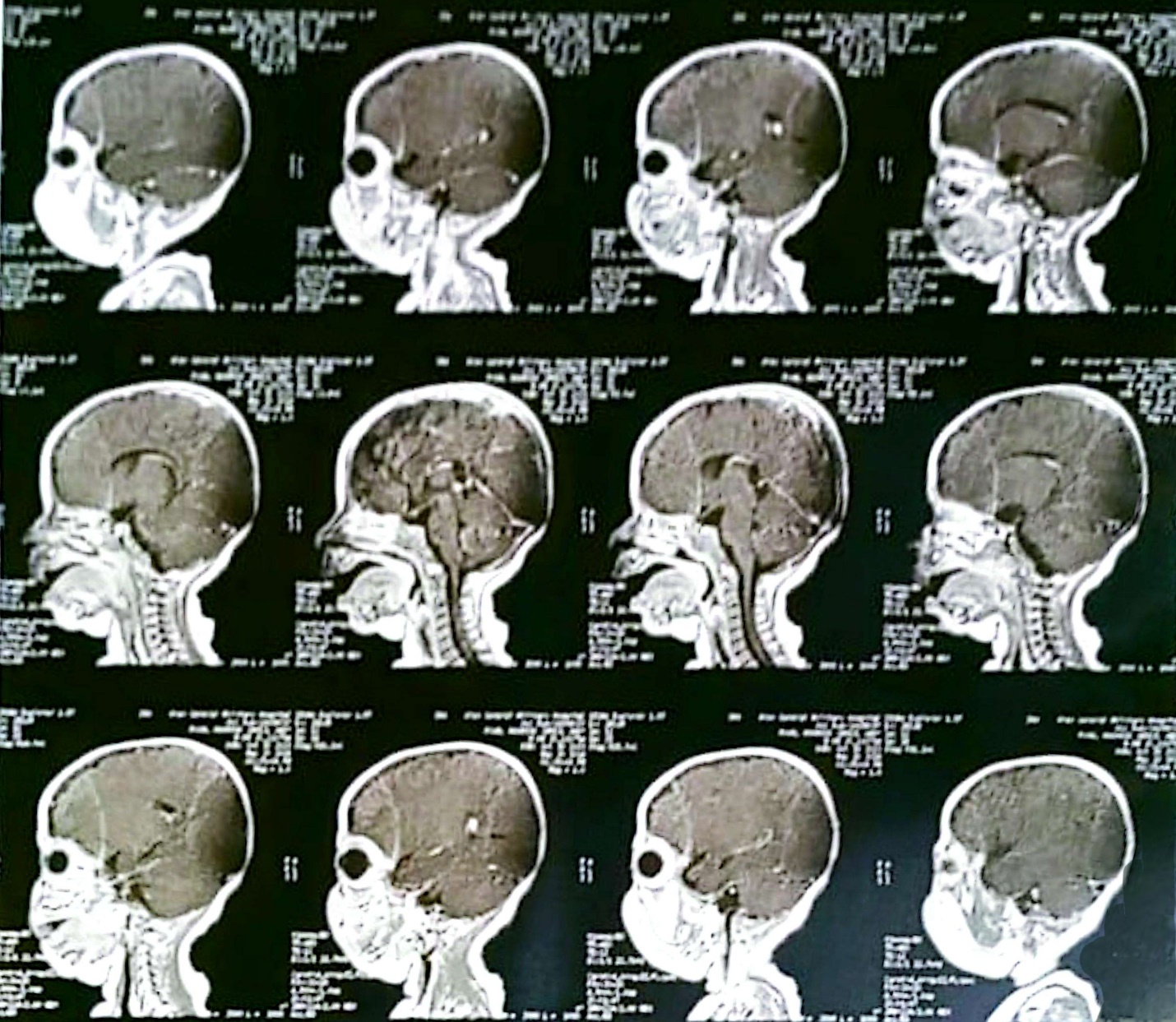


**Supplementary Figure.2 – (**Sagittal views of patient’s brain MRI with contrast)


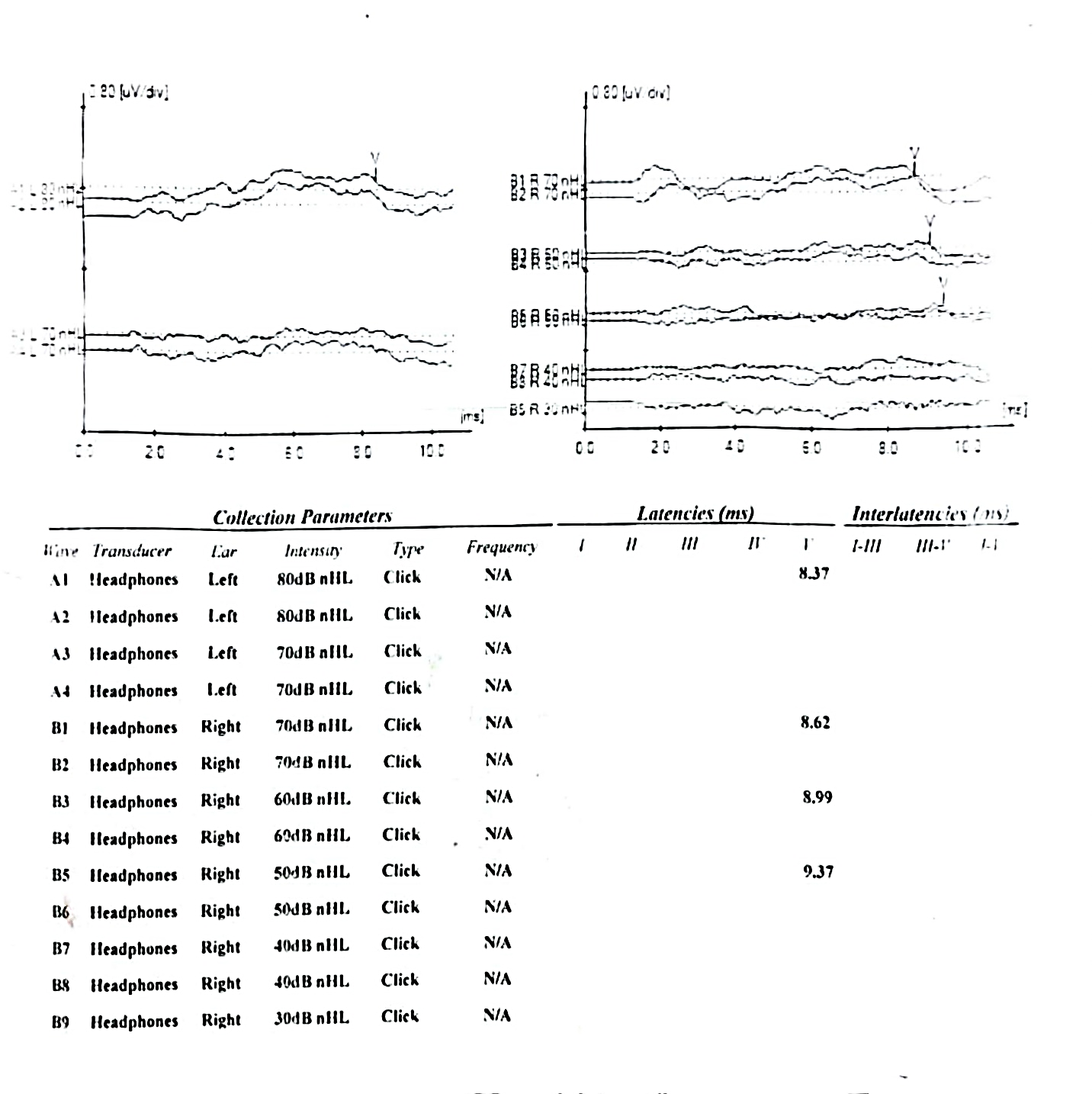


**Supplementary Figure.3 –** (Evoked potential report of the auditory brainstem response test)

| Supplementary Table.1 – Differential Diagnosis of ASMD Type A/B | | | |
| --- | --- | --- | --- |
| Feature | **ASMD Type A/B** | **Gaucher Disease** | **Tay-Sachs Disease** |
| Enzyme Deficiency | Acid sphingomyelinase | Glucocerebrosidase | Hexosaminidase A |
| Gene | *SMPD1* | *GBA* | *HEXA* |
| Accumulated Substrate | Sphingomyelin | Glucocerebroside | GM2 ganglioside |
| Hepatosplenomegaly | Yes | Yes | No |
| Neurological Involvement | Yes | Rare | Yes |
| Cherry-Red Spot | Common | No | Yes |
| Bone Pain/Lesions | No | Yes | No |
